# A bone-conducted Tullio phenomenon—A bridge to understand skull vibration induced nystagmus in superior canal dehiscence

**DOI:** 10.3389/fneur.2023.1183040

**Published:** 2023-06-09

**Authors:** Georges Dumas, Ian S. Curthoys, Andrea Castellucci, Laurent Dumas, Philippe Perrin, Sébastien Schmerber

**Affiliations:** ^1^Department of Oto-Rhino-Laryngology, Head and Neck Surgery, University Hospital, Grenoble, France; ^2^EA 3450 DevAH–Development, Adaptation and Handicap, Faculty of Medicine, University of Lorraine, Nancy, France; ^3^Vestibular Research Laboratory, School of Psychology, The University of Sydney, Sydney, NSW, Australia; ^4^ENT Unit, Department of Surgery, AUSL–IRCCS di reggio Emilia, Reggio Emilia, Italy; ^5^Laboratoire Radiopharmaceutiques Biocliniques (LRB), INSERM U1039, Faculté de Médecine La Tronche, Université Grenoble Alpes, Grenoble, France; ^6^Department of Paediatric Oto-Rhino-Laryngology, University Hospital of Nancy, Vandovuvre-lés-Nancy, France; ^7^Brain Tech Laboratory, INSERM UMR 2015, Grenoble, France

**Keywords:** Tullio, skull vibration induced nystagmus, minor syndrome, anterior canal, nystagmus, vertigo, bone conduction

## Abstract

Nystagmus produced in response to air-conducted sound (ACS) stimulation—the Tullio phenomenon—is well known in patients with a semicircular canal (SCC) dehiscence (SCD). Here we consider the evidence that bone-conducted vibration (BCV) is also an effective stimulus for generating the Tullio phenomenon. We relate the clinical evidence based on clinical data extracted from literature to the recent evidence about the physical mechanism by which BCV may cause this nystagmus and the neural evidence confirming the likely mechanism. The hypothetical physical mechanism by which BCV activates SCC afferent neurons in SCD patients is that traveling waves are generated in the endolymph, initiated at the site of the dehiscence. We contend that the nystagmus and symptoms observed after cranial BCV in SCD patients is a variant of Skull Vibration Induced Nystagmus (SVIN) used to identify unilateral vestibular loss (uVL) with the major difference being that in uVL the nystagmus beats away from the affected ear whereas in Tullio to BCV the nystagmus beats usually toward the affected ear with the SCD. We suggest that the cause of this difference is a cycle-by-cycle activation of SCC afferents from the remaining ear, which are not canceled centrally by simultaneous afferent input from the opposite ear, because of its reduced or absent function in uVL. In the Tullio phenomenon, this cycle-by-cycle neural activation is complemented by fluid streaming and thus cupula deflection caused by the repeated compression of each cycle of the stimuli. In this way, the Tullio phenomenon to BCV is a version of skull vibration-induced nystagmus.

## 1. Introduction

### 1.1. The Tullio phenomenon

Tullio found that following the fenestration of one semicircular canal (SCC) of a pigeon, ACS stimuli elicited pendular movement of the body in the plane of the opened SCC and, at higher intensity, ocular nystagmus. He showed that the sound stimuli produced fluid displacement of endolymph corresponding to these responses. Of the three SCCs in a labyrinth, it was the fenestrated SCC that had the largest displacement of endolymph and so caused the eye movement in the plane of that canal (1, 2). A co-stimulation of the utricular macula was suggested since Tullio also noted that when a tuning fork was close to the pigeon's ear, the membranous wall of the utricle oscillated.

In human patients, nystagmus due to ACS is found in a number of pathologies (3) and it has been observed in unilateral SCD patients (uSCD) using high-intensity ACS stimulation 120 to 160 dB SPL (4–7). We have recently observed that similar nystagmus can be provoked by BCV stimuli applied to the cranium using frequencies of between 100 to 800 Hz in uSCD patients (8). The vertex location was particularly effective in inducing nystagmus which was associated with reports by the patients of dizziness (8–10). The important connection between ACS and BCV is that after making artificial SCD, single SCC neurons can be activated by both ACS and BCV up to very high frequencies (>3,000 Hz) (11, 12). To clarify the link between SVIN to BCV and Tullio to BCV, the questions we address are the extent to which BCV is an efficient way of inducing a Tullio phenomenon in uSCD patients, the direction of the nystagmus, its probable physical basis, the neural mechanism, and the cause of the variable responses within and between patients.

### 1.2. Clinical evidence

PubMed/Medline and Scopus were searched for the 2 last decades (2005–2022) and selection was performed according to the procedure and policy indicated by Preferred Reporting Items for Systematic Reviews and Meta-Analyses (PRISMA) guidelines. The search criteria were “Vibration induced Nystagmus” (all fields), cranial vibrations (all fields), and Superior canal dehiscence (all fields). Only SCD patients corresponding to the definition given by Minor et al. (6) and with characteristics summarized by Ward et al. in 2017 (7) (stated below) were considered and included.

The initial flow chart was 12 publications.

Patients met the following conditions proposed as diagnostic criteria for superior canal dehiscence syndrome (7):

1. High-resolution computed tomography images (≤0.625-mm slice thickness) reformatted in the plane of the superior SCC demonstrating dehiscence.
2. At least one of the following symptoms consistent with SCDS: A. Bone conduction hyperacusis (in the form of autophony, audible eye movements, audible footsteps, etc.) B. Sound-induced vertigo C. Pressure-induced vertigo (*via* nasal or glottic Valsalva or pressure applied to the external auditory canal) D. Pulsatile tinnitus.
3. At least one of the following diagnostic tests indicating a third mobile window A. Negative bone conduction thresholds for low frequencies on pure tone audiometry B. Enhanced VEMP responses (low cervical VEMP thresholds or high ocular VEMP amplitudes) C. Elevated ratio of the summating potential to action potential on electrocochleography in the absence of a sensorineural hearing loss.

Three out of the 12 publications were not included because they did not meet the inclusion criteria and/or were an unacceptably small series of patients (<5).

SVINT results in SCD patients described in the literature (8, 9, 13–18) following these criteria are reported in Table 1.

**Table 1:** Skull vibration induced nystagmus (SVIN) characteristics in superior semicircular canal dehiscence, Literature data.

	**Se (%)**	**Cohort size**	**Patients count uSCD/ bSCD**	**Stimulus locations Optimal location**	**Stimulus Frequency (Hz)**	**SHC num (*%*)**	**SVC num (*%*)**	**STC num *(%)***	**SVIN DCF Location num (*%*)**	**SVIN DCF Frequency num (*%*)**	**After Nyst num**	**AC Tullio num (*%*) Hennebert num (*%*)**
White et al. 2007 (13)	100	8	6	RM, LM, Vx, SO	100	ND	Dw 2 Up 1	Contr 3 Ipsi 2	1/8 (*12*)	ND	1/8 combined with positional nyst.	ND 2/8 (*25*)
			2	Opt. SO		ND	Dw 1 Up 0	2				
Schmerber et al. 2008 (17)	-	6	4	RM, LM Vx	100	Ipsi 3 (*50*)	Dw 2 Up 2	ND	ND	ND	ND	rarely
			2	Opt Vx	-	0	0	ND				
Manzari et al. 2008 (15)	100	16	97	RM, LM -	100	Poor Poor	Dw 7 Up 2Dw 4 Up 1	Ipsi 4 Contr 0	HC: 7/9 VC: 7/9 TC: 6/7	ND	ND	9/16 (*56*) ND
Aw et al. 2011 (14) (ViVOR)	100	17	12	RM, LM	500	Small (negligible)	ViVOR up	ViVOR contr	0	ND	ND	9/12 (*80*) ND
			5	-	-	Small	ViVOR up++		TC: RM/LM			5/5 (*100*) ND
Dumas et al. 2014 (9)	82	17	17	RM, LM Vx Opt Vx	100	Ipsi (*70*)	Up (*47*)	Ipsi (*62*)	HC 2/17 VC 1/17 TC 2 /17	ND	4/17	4/17 (*24*) ND
			0	0		0	0	0				ND
Park et al. 2014 (16) (ViVOR)	90	10	9	RM, LM	100	ViVOR Ipsi (*18*) Contr (*70*)	Dw (*21*)Up (*75*)	1	ND	ND	ND	8/10 (*80*) ND
			1	-	-	-	-	-				
Mehta et al. 2015 (36)	55	38 cSCD (50 cSCD+nSCD)	ND	RM, LM	ND	ND	ND	ND	ND	ND	ND	10/38 (*26*) 8/38 (*21*)
Dumas et al. 2019 (8)	86	40	27	RM, LM, Vx	30–800	Ipsi *(85)* Contr *(12)*	Dw (*40)* Up *(60)*	Ipsi *(57)* Contr *(2)*	2/10 (*20*)	2/8 (*25*)	3/15	5/20 (*25*) ND ND ND
	54		13	Opt Vx			Dw *(25)*Up *(75)*					
Batuecas et al. 2022 (18)	66	30	25	RM, LM, Vx	100	6/30	Up 14/30	ND	ND	ND	ND	ND ND ND ND
			5	Opt ND		4/9	Up 4/9	ND				

Stimulus location: RM, Right mastoid; LM, Left mastoid; Vx, Vertex; SO, suboccipital; Opt, optimal location; Nystagmus direction: ipsi: SVIN beating ipsilaterally; contr: SVIN beating contralaterally; For the torsional component, SVIN direction is given as follows: an ipsilateral beating nystagmus corresponds to the fast mobilization (jerk) of the eye upper part directed toward the patient lesion. A contralateral beating nystagmus corresponds to the fast mobilization (jerk) of the eye upper part directed away from the patient lesion (or toward the intact side). ViVOR, vibration induced Vestibulo Ocular Reflex. In this last condition the directions mentioned indicate the direction of the eye slow phase displacement. uSCD, unilateral superior canal dehiscence; bSCD: bilateral superior canal dehiscence; DCF, Direction Changing Following; SHC, SVIN horizontal component direction; SVC, SVIN vertical component direction; STC, SVIN torsional component direction; HC, Horizontal component; TC, Torsional component; VC, vertical component; ASN, after stimulus nystagmus; ND, no data; cSCD, complete SCD; nSCD, near dehiscent SCD.

### 1.3. Bone conduction mechanisms

At present the possible mechanisms for BCV inducing a Tullio phenomenon are (as summarized by Stenfeld et al.) (19, 20):

1. Sound radiated into the ear canal.
2. Inertial effect of middle ear ossicles.
3. Inertial effect of the fluid in the inner ear.
4. Space alteration of the inner ear due to vibrations of the bone surrounding the inner ear (compression of the cochlear wall).
5. It has been suggested that sound can reach the inner ear by vibrations applied to non-osseous factors (soft tissue, fluids). This last contribution particularly via the cerebrospinal fluid (CSF) through the cochlear aqueduct is very poor, and negligible in normal subjects but is likely substantial in uSCD patients depending on the location stimulated by vibration.

## 2. Discussion

The insight of SVIN in clinical practice is to contribute to SCD diagnosis as a useful and understandable tool when interpreted in the light of a bone-conducted Tullio phenomenon (BCTP).

A “Nystagmus produced by mastoid vibration in patients with Tullio's phenomenon” was described in 1998 by Cremer, Zee, and Minor at the XX^th^ Regular Meeting of the Barany Society (21). These authors suggested for the first time that there is a link between SVINT and the Tullio phenomenon. However, there is one major difference: in patients with uVL the SVIN nystagmus beats away from the affected ear whereas in SCD patients the SVIN beats usually toward the affected ear. The nystagmus to cranial BCV in SCD patients has torsional and horizontal components (9, 15, 16, 22, 23) and is most often a vertical down-beating nystagmus (10, 13, 14) without after-nystagmus. BCV activates both canal and otolith structures (12).

The results in SCD patients can be attributed to the direct stimulation of the vestibular hair bundles and irregular afferent neurons on the side of the SCD (11). The excitatory type of nystagmus response corresponds to the BCV causing semicircular canal activation on the side of the dehiscence (24). This effect has been observed in clinical practice (8, 9, 16) (Table 1): SVIN torsional or horizontal components beat ipsilaterally in 82% of cases after vertex stimulations in series from Dumas G et al. (8, 10). However, in a few cases, apparent discrepancies concerning the nystagmus direction following the stimulus location (10, 13, 23) and frequency (direction changing nystagmus) (8, 10) have been described, and rare cases of prolonged after-nystagmus mimicking a “spontaneous nystagmus” after the stimulus offset have been reported (8, 10). We suggest that these last results need to be interpreted in light of the flow and pumping mechanism of the Tullio phenomenon discussed below (25).

- The variability of the direction of the vertical component in previous studies (Table 1) (up or down beating nystagmus) depends sometimes on stimulus location (8, 10, 14, 15) but also sometimes on frequency (8, 10). Recent data reported in an oral communication in SCD patients presented by Dumas et al. Barany Society, 2022 (10) showed that in 20% of cases, the direction of the vertical component changed when stimulus location changed (i.e. vertex vs. mastoids or right mastoid vs. left mastoid). Furthermore, in 25% of patients stimulated in one location, the nystagmus direction depended on stimulus frequency (100 vs. 700 Hz). The vertical component was up-beating by 66 % on mastoid BCV stimulation. In 14% of patients, a primarily vertical down beating nystagmus was observed when the vertex was stimulated. The nystagmus was enhanced when the gaze was directed toward the plane of the dehiscent superior SCC or of the most dehiscent or most symptomatic side (hearing loss) in the case of asymmetric bilateral SCD (bSCD) (8, 10). The 3 D recording analysis in 21 patients with a positive SVIN showed that the slow phase eye velocity (SPV) of SVIN of the vertical component after vertex stimulation was higher in bSCD (6.33 ± 5.13°/s) than in uSCD (2.14 ± 3.18) (*p* < 0.05) (10). A similar increase of the vertical component to short-duration ACS clicks has been described by Aw et al. (14) in bSCD.

### 2.1. What is the physical mechanism, which allows BCV to activate canal receptors in SCD

Grieser et al. (26) reported the results of a simulation using a computer model of fluid flow in a superior vertical semicircular canal with an artificial SCD. They found that ACS stimulation caused a unidirectional streaming flow of endolymph which was ampullofugal (and thus excitatory). The authors likened this to the fluid flow generated in a valveless impedance pump (i.e., the Liebau principle) (27) where repeated compression of a flexible tube causes unidirectional movement of the fluid within the tube, and the direction of fluid movement depended on where the compression was located amongst many other factors. Grieser's results imply that in a human patient with a dehiscence of the superior canal, such repeated compressions (i.e., cycles of the stimulus), would generate a cupula deflection in an excitatory direction and so result in down-beating nystagmus. This is the direction found in many patients. However, some neural data did not accord with the Grieser model. Carey et al. (28) measured single canal neuron responses after an artificial SCD in chinchillas and found that the neural response to ACS reversed at different frequencies of the ACS stimulus, implying that the direction of endolymph flow reversed at particular frequencies. Frequency-dependent reversal of endolymph flow cannot be accommodated by the Grieser model.

Iversen et al. (25) developed a model with the difference being that sound or vibration caused traveling waves of the endolymph in the dehiscent canal to be generated in the endolymph. These waves also caused cupula deflection but with the important difference, compared to Grieser et al., that the flow direction depended on frequency in accord with the neural data—and it reversed at particular frequencies.

Iversen et al. then actually measured the fluid flow in a toadfish SCC and confirmed their model predictions: that sound energy causes fluid flow at the location of the defect, giving rise to traveling waves that subsequently excite mechano-electrical transduction in the vestibular sensory organs. They showed the direction of the fluid flow to BCV stimulation depended on the location of the stimulus, the size of the dehiscence, and the frequency of the stimulus. They also recorded single semicircular canal neurons (in toadfish) which confirmed the predictions from their measurements and modeling. They observed an excitatory endolymphatic ampullopetal flow in the horizontal SCC at frequencies from 100 Hz to around 500 Hz and a reversal of the flow and inhibition of neural regular discharges at frequencies from 500 to 850 Hz. In short, the “traveling wave” model of endolymph flow after artificially created SCD is an excellent account of the mechanism of semicircular canal response to BCV after SCD, both of the physical mechanisms and the neural results and thus the human patient nystagmus. In humans, such a reversal was observed at frequencies around 300 Hz or in rare cases at 30 Hz (10).

### 2.2. Other VIN clinical data explained in the wake of the BC Tullio concept

It was initially proposed that the horizontal component of SVIN observed in SCD was negligible (13, 14). That result is in agreement with the results of Aw et al. who studied the vibration-induced vestibulo-ocular reflex with scleral coils. However in the study by Aw et al. patients were in a supine position, head fixed, and viewing a stationary target at 600 mm (fixation usually inhibits peripheral nystagmus horizontal component), and the stimuli were of very short duration.

Under more realistic clinical conditions, a noticeable horizontal component was found in uSCD patients with ocular fixation denied and these patients showed an ipsilaterally beating nystagmus on vertex stimulation Dumas et al. (8, 9, 22)—that is, the quick phases beating toward the ear with the SCD. Horizontal components were also reported by Koo et al. as a consistent response (11.4°/s) (29) and more recently by Park et al. (16). This suggests either the contribution of the horizontal SCC afferent neurons as proposed by Park et al. or possibly the contribution of utricular activation as suggested by Dumas et al. (8, 10), Halmagyi et al. (30). The canal origin is more likely because Iversen et al. showed a “spread” of activation from the canal with the SCD to the horizontal canal.

The initial description of Tullio noted a concomitant utricular stimulation. In clinical practice this concomitant utricle stimulation is well corroborated by vestibular tests: the oVEMP (and also cVEMP for mainly the sacculus) shows a strong response (higher amplitudes and lower threshold) in SCD (31).

**Figure 1:**
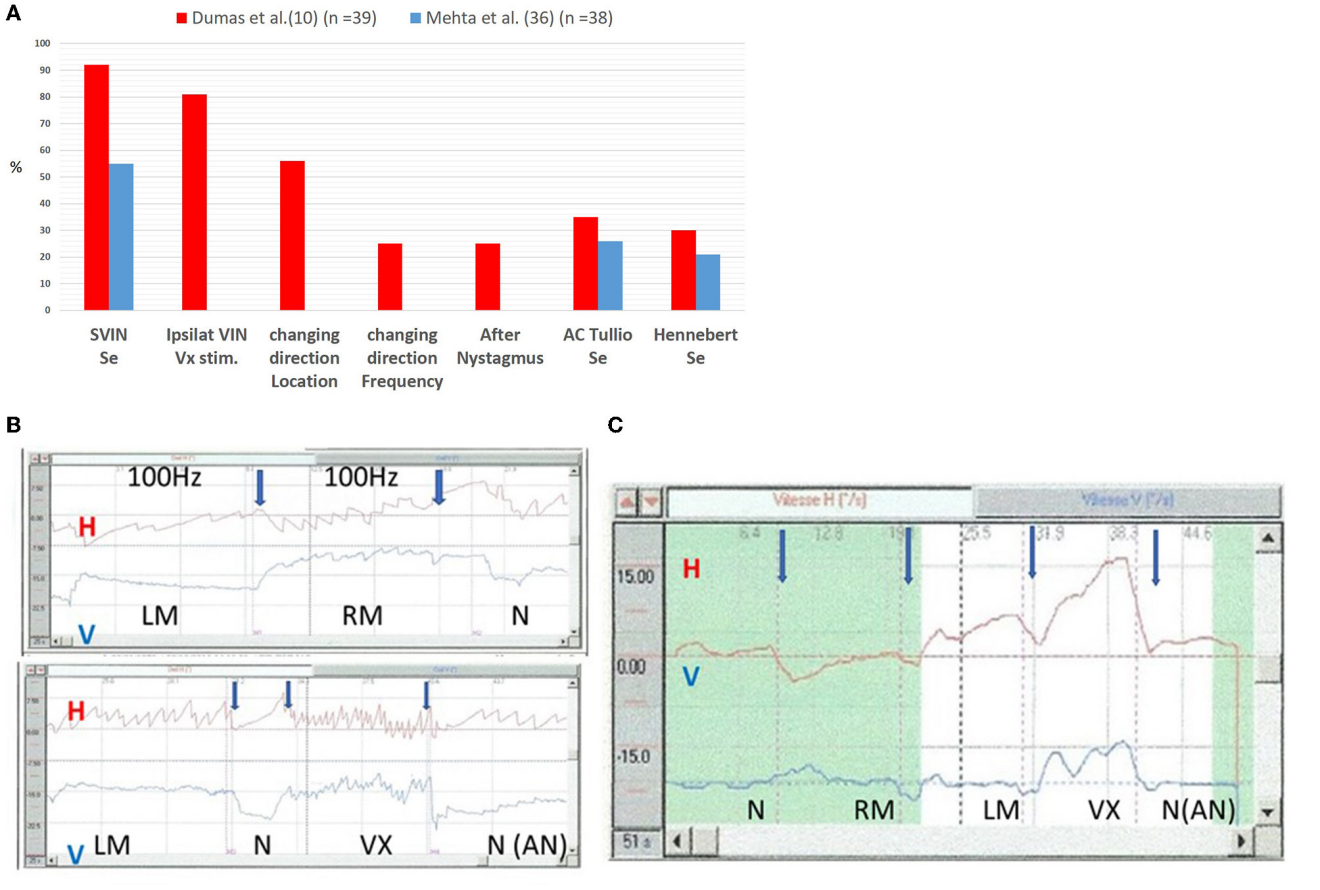
Sensitivity of SVIN in SCD as compared to Air conducted Tullio phenomenon and Hennebert sign. **(A)** Sensitivity and characteristics. Ipsilateral VIN. Vx Stimulation: % of positive SVIN ipsilaterally beating toward lesion after vertex Stimulation. Changing direction location: Changing direction following stimulus location (Vx stim vs. Mastoids; RM vs. LM). Changing direction frequency: changing direction VIN following stimulus frequency: this analysis was performed in 12 uSCD. The stimulation (Bruel & Kjaer Minishaker, Naerum, Denmark) was performed at frequencies from 30 to 800 Hz. **(B, C)** Time series of Horizontal and Vertical eye movements of a patient with a left unilateral SCD in response to 100 Hz vibration at different skull locations. **(B)** *Direct recordings* at 100 Hz on LM, RM, and vertex (VX) locations: responses are higher on VX stimulation. The horizontal component direction is different for right mastoid (RM) stimulation (quick phases of SVIN beat toward the right side) vs. left mastoid (LM) stimulation (quick phases of SVIN beat toward the left side) and for vertex stimulation (quick phases beat toward the left side). A moderate horizontal after nystagmus (AN) (4°/s) is observed after VX stimulation. The vertical component observed on vertex stimulation in this example is down beating. LM, left mastoid stimulation; RM, right mastoid stimulation; Vx, vertex stimulation. **(C)** *Slow phase velocity* (SPV) recording at 100 Hz: order of stimulation: RM, LM, and then Vertex (VX) The direction in response to stimulation on Vertex is ipsilaterally beating (quick phases to the lesion side) and the slow phase velocity is higher than in response to mastoid stimulation. After stimulation offset, an after nystagmus (AN) of the horizontal component is recorded. SVIN, Skull Vibration Induced Nystagmus; Vx, vertex; RM, right mastoid; LM, left mastoid; uSCD, unilateral superior canal dehiscence. ACTP, air conducted Tullio phenomenon; BCTP, Bone conducted Tullio Phenomenon; Se, sensitivity; AC Tullio, air conducted Tullio phenomenon.

The strong efficiency of vertex stimulation in SCD patients contrasts with the usual inefficiency of vertex stimulation in patients with unilateral vestibular loss (UVL) (8, 9). This may be because the vibration at the vertex is more directly transmitted by compressive waves through the brain and cerebrospinal fluid to the endolymph via the middle fossa fistula constituted by the dehiscence. This has been suggested by Sohmer et al. (32, 33), Brantberg et al. (34), and Freeman et al. (35). In SCD patients SVIN is more often observed for vertex BCV stimulation when performed after mastoid stimulation (SVIN is observed in 82 % of positive patients on Vx) (Figure 1B) (10) than when the stimulus order protocol stimulates first the vertex and then the mastoids (in this last condition the 2 locations of stimulation are as efficient and an SVIN is obtained for each in 50% of cases). This can be explained by the larger cupula deflection to a longer stimulus duration caused by fluid streaming together with the repeated compression of each cycle of the stimuli. In clinical practice an after-nystagmus is observed in 25% of cases. This observation remains rare possibly because the traveling wave and the flow-induced at the level of the dehiscence are not always strong enough to provoke a sufficient deflection of the cupula (and the response of inner ear vestibular hair cells) but the vibration is sufficient to only directly activate the hair bundle of type I inner ear hair cells (cycle-by-cycle phase-locked activation of action potentials) favored by the BC facilitation toward the lesion side.

BCV in SCD constitutes a more efficient test than AC Tullio phenomenon (ACTP) in clinical practice.

Mehta et al. (36) (Figure 1) observed in 38 definite SCD patients a positive Hennebert sign, an ACTP, and a positive SVIN in 21%, 26%, and 55% of cases, respectively. A positive SVIN in SCD is described in 62 % (18), 82% (11), and 88% (8) of cases, and even 100% of cases in a few series (13, 15). These results point to possibly higher sensitivity and efficiency of a BC Tullio phenomenon (BCTP) as compared to an ACTP.

SCD patients with a positive SVINT most often report dizziness or vertigo or discomfort during skull stimulation (8, 17, 23). Dumas et al. (8) reported dizziness and nausea in 60% of SCD (16/27 patients) when stimulation is repeated, similar to Minor et al. (6) and Ward et al. (7) with ACTP.

## 3. Conclusion

This paper shows the new insight of SVIN in clinical practice among other vestibular explorations in SCD patients when it is interpreted as a bone-conducted Tullio phenomenon.

The Tullio phenomenon initially described after air-conducted sound stimulation is currently most often observed in SCD patients but is not specific to this stimulus. The very similar symptoms of dizziness and nystagmus provoked by BCV stimulation are often observed in SCD and appear to constitute a more efficient stimulus compared to air-conducted sound stimulation to elicit a Tullio phenomenon.

SVINT in SCD patients stimulated by BCV is comprised of a vertical and torsional component generated by the superior canal and by a horizontal component caused by spread to the horizontal SCC or by a utricular contribution. The direction of SVINT in SCD patients is most often beating ipsilaterally (when vertex is stimulated) but nystagmus direction cannot be always predicted in any simple fashion because of the instability of the created endolymphatic flow provoking a stimulation of inner ear hair cells of the cupula as shown by Iversen et al. This instability explains the variability following stimulus location and frequency observed in some patients but also discrepancies between investigators.

## Data availability statement

The original contributions presented in the study are included in the article/supplementary material, further inquiries can be directed to the corresponding author.

## Author contributions

GD and IC: original idea, general coordination, review, and co-writing the article. PP and SS: contributed to the MS editing and discussion. AC and LD: contributed to the table editing and figures. All authors contributed equally to the rewriting of the article and its conclusions.
